# Cataphoresis

**Published:** 1897-03

**Authors:** Cyrus A. Allen

**Affiliations:** Buffalo.


ARTICLE II.

### CATAPHORESIS.

BY DR. CYRUS A. ALLEN, BUFFALO.

Read at joint meeting of Buffalo and Toronto Dental Societies, Niagara-on-the-Lake, July 18, 1896.

In the hurry and rush of a daily practice, large as I know we all possess, it is difficult to introduce radical changes in methods. This I believe to be particularly true when it comes to the acceptance or even consideration of methods savoring of the "painless" character. However, the busy man must not forget that science progresses and must be considered, and that all who would "be in at the finish" must conform themselves to its changing condition. This declaration should not be construed in any manner as urging the endorsement of every new claimant. I think you will agree with me that even in our noble profession there are often pretensions to truth and fact which experience never can verify. However, failure along lines of declared truth should not close our eyes to the possibilities of advancement.

Ever since my earliest recollections I have been accustomed to the atmosphere of dental operations; and I must confess, with all courtesy to my good father, who was a pioneer in our art, and who was always *inter primos* in his life-work, that those early memories were not of the nature to make a boy respect his father. However, as time has gone on, we realize that he was even of the first, and that the professional crudities then extant were largely responsible for the degree of respect in which our art was held in that early day.

The general public has so loved the results of our labors, no matter by what discomforts received, that to-day we have in America alone about 25,000 dentists, with more to follow, judging from the liberal inducements offered by colleges to possible students in this field. But with advances made in other scientific and professional fields in recent years there has come a popular demand for "painless dentistry," which to most of us is a synonym for quackery—something alluring to the public. For the benefit of humanity and the good name of our profession, I would that the adjective "painless" had been omitted from our language. However, its existence and disgraceful application may have been in some small measure of use to our profession; for if its profligate use, employed to bunco the public, has awakened us, then is it an instrument of good.

An era of quackery and charlatanism has been abroad among us; ignorance and cupidity, with their train of misapplied energies, have produced results dangerous and often fatal to a public ever ready to try some "new thing." The fatal, to say nothing of harmful results following the injections of various secret preparations for painless operations, compel the conservative mind to hesitate when any new scheme is presented to alleviate human suffering. However, I believe from my own experience, added to the clinical demonstrations of trusted friends, that cataphoresis opens a field wide and useful in dentistry, and absolutely free from the lurking dangers which always accompany the use of unknown and secret preparations.

Let us consider some of the more conspicuous designations for cataphoric treatment in our special field: (1) Its employment in sterilizing medications in roots, etc. (2) Arresting abscesses, incipient or otherwise, by the iodine treatment. (3) For obtunding sensibility previous to the lancing of abscess after pus formation. (4) For the treatment of pyorrhœa alveolaris. (5) For the treatment of acute pericementitis. From my own clinical experience the application of this treatment has a wide, and at present little understood usefulness, (6) For bleaching of discolored teeth. (7) For the obtunding of sensitive dentine preparatory to the insertion of fillings, an application which at once appeals to all practicing dentists. (8) For the preparation of teeth and roots for crown adjustment—another wide field of usefulness. (9) For the immediate extirpation of inflamed, congested and diseased pulps. (10) And others without number, according to the development of future experience.

May it not be well here to define cataphoresis? This modern term comes from the two Greek words, "cata" and "phorein"—"cata" with its signification, downward, and "phorein" to bear, to travel. Collectively, then, these two words would mean in general application, 'travelling downward." But in its application as we are employing it to-day, why should we say downward and not upward? This may be explained from the fact that the older electricians possessed themselves of the idea that there was a positive potential which was always upward, and a negative which was always downward.

The term "cataphoresis" was used later on to express the more special application of the phenomena to tissue. We may then abruptly define cataphoresis as "flow of fluids from the positive to the negative pole." If this be true you will readily agree with me that if these fluids should contain elements in solution which would make them medicines, these medicines must flow from the positive to the negative conveyed by the current; and that if you make application to the one tissue by the positive pole and any remote part by the negative, and this solution should have a tendency to flow with the current, then these medicines will travel in that direction.

To what extent then do remedies under this influence travel? This is a matter which must be left largely to future demonstration. So far as I am personally concerned, I know absolutely nothing in regard to it of a definite character. Some good authorities, however, claim that if you apply to one side of elevated tissue (as the alveolus) the negative pole, and to the other the positive pole, loaded with a solution of cocaine, the anæsthetic effect will only reach half-way through that tissue. More than this, that the remaining half will not only not be influenced by the cocaine, but will also be rather increased in hypersensitiveness.

It will be well here to discuss somewhat the mechanism of the apparatus, for, simple as it is, you should have some definite knowledge of its operation. I will call your attention to (1) The voltage which concisely represents the pressure of the current, as indicated by the numbered attachments. May increase at pleasure. (2) The milliampere dial records simply the flow of the current through the tissue. A mechanism attached to the apparatus, which is designated as the current controller, is intended simply to furnish in the smallest quantities further pressure upon the tissue as may be suggested by the case in hand.

We now come to the question of current strength which may be required. My clinical experience seems to suggest that a large voltage is not at all necessary in securing the desired results. Indeed, so firm am I in this conviction, that I boldly assert that from one half to one and a half milliampere registrations will be sufficient to anæsthetize perfectly almost every case which may present itself. Another very important reason why a low voltage should be applied is for the comfort of our patients, which we should under no circumstances lose sight of.

Another very important element in the success of this method consists in the *uniform* and *continuous constancy* of current application. If the current be spasmodic, interrupted or unreliable in any degree, it will not only impair the results sought, but will also seriously disturb the comfort of the patient. There is nothing which so quickly develops remonstrance from the one operated upon as an interrupted current or the sudden elevation from a low to a high voltage. To this general rule, emphatic as I would make it, there are some exceptions which do not figure in the general summing up. The importance of continuous application of current should be borne in mind even to the end, that when a renewed application of the anæsthetic solution is desired, no disconnection should take place, but rather that the solution should be added with appliances *in situ*, in order to avoid the disagreeableness of a broken current and its reapplication.

We have indefinitely referred to solutions and anæsthesia in general without any special reference to what these solutions should contain. I should lay it down as an emphatic law of necessity that all cocaine solutions should be prepared for each individual case, for the reason simply that with certain atmospheric conditions only a few hours may suffice to absolutely destroy their efficacy. The leading chemical laboratories of the country prepare in tablet form many of the remedies used by us in such attenuated quantities that the most bland solution may be prepared by even the novice, simply by reading the directions on the preparation. A method employed by me for cocaine anæsthesia is to place upon the marble slab a small quantity of the pulverized crystal which may be readily picked up by a piece of cotton of the proper size well moistened, and then carried directly to the cavity. The previous moistening of the cotton will furnish a sufficient medium for the conduction of the current, together with the alkaloid, to the place desired.

Some days since, in the use of the agent, I added a mild solution of chloride of sodium, or common salt, upon a suggestion which I received from a recent publication.

The result in this case, which was a badly inflamed pulp aching continuously for the past eight hours, was most happy, as the current contact continued only for nine minutes, which produced a condition whereby I was allowed to remove the thin lamina of dentine overlaying the pulp, and immediately thereafter proceeding to the successful removal of the pulp itself with scarcely any pain to the patient. This case, I learn from my record book, gave me the quickest and happiest result which I have enjoyed since employing this method. Whether the result was directly dependent upon the addition of the salt solution I am unable to state, but am inclined to the belief that a cocaine solution is not altogether a perfect conductor of the electrical fluid, and if this be so it suggests to us very forcibly that there should be always an addition made to our solutions which will render them perfect conductors.

In regard to the application, it may be briefly stated that the positive pole, which is indicated by the star upon the apparatus, should always be applied directly to the tissue upon which the operation is to be performed. The application of the electrode may be either upon the same or the opposite side of the anatomy, and from my own clinical experience there seems to be no preference in the matter, although it has been authoritatively published that the applications of the positive and the negative should be upon opposite sides. The manner of application to any given cavity will be suggested by the ingenuity of the operator himself, only bear in mind that he shall have perfect contact with the solution contained in the cavity of the tooth. The varying cases which may present themselves will suggest differently shaped appliances, which may be made upon a moment's notice with pliers and wire of suitable gauge.

Anæsthetizing the soft tissues upon any part of the anatomy may be readily accomplished by the application of the agent; held firmly in position by the cup-shaped device furnished by the manufacturer, or any which you yourself may prepare. It is hardly necessary to go minutely into the details of each case, but rather is it better that these minor affairs should be left entirely to your own judgment.

While I regard this new method in our hands as a means of ameliorating human suffering and thereby tending greatly toward the removal from our profession of many traditions of horror, I would urge a word of warning against its unintelligent use. This warning word is particularly directed toward those of limited experience in the treatment of certain lesions of the teeth. Labial and buccal cavities are here our greatest concern. With absolute immunity from pain in an extensive labial cavity, I fear that the unwary or inexperienced may bring this good device into discredit by the wholesale destruction of pulps by virtue of too free removal ot tissue and placing a filling of perfect conductibility in dangerous proximity to these organs.

*Toxic* results I know not of. Intercourse with my professional friends, and reports in our dental journals, relate no fatalities from this new departure. Nor do I learn from friends or from my own clinical experience that there is even the slightest systemic disturbance such as might be expected upon liberal cocaine absorption. Whether this agent enters into the tissue only in a circumscribed area, or whether its absorption systematically is prevented through some subtle influence of the electrical fluid, I am wholly unprepared to state. But the fact remains that I have yet to observe any systemic derangement from my own experience.

It may be asked if we claim perfect results from cataphoric treatment in all instances. By no means; on the contrary, my case book presents a total of widely varying results all the way from absolute failure to the most exalted success. In a careful study of these records I do not find any suggestion that individual temperament has been a dominating influence. This observation, I believe, will hold good only so far as it does not include those magnified extremes where Nature seems almost to present anomalous conditions. Magnified temperaments frequently furnish peculiar clinical phenomena, often erratic and even ungovernable by the usual methods. We all are aware that individuals vary greatly in their physical capacity for the electric current, while outwardly there may be no manifest difference in temperament; therefore, we may conclude that the individual nervous organization, with its capacity to repel or welcome the medicated current, will largely determine the clinical results. For it musc be constantly borne in mind that anæsthesia can only be produced after and by the influence of the current's passage through the tissue, accompanied by the proper agent. The simple application of the current and agent to the desired parts can avail nothing in themselves. The current and agent must absolutely pass through the tissues. When this takes place, even to the minute extent of a fractional mill, the dial will indicate the fact. Ordinarily the deflection of the needle will be immediate, but not always, for if the case in hand be one that so curiously repels the electrical fluid—through some unknown constitution of its own—then we may expect no movement of the needle. I find these cases the most difficult to anæsthetize, having consumed one hour and forty-five minutes in one instance in the removal of a pulp. The happiest result which I have recorded was complete anæsthesia of a pulp in seven and a half minutes, the period of application being usually ten to fifteen minutes in my experience — the time required for the relief of cavity sensitiveness being on an average about half that required for the pulp, and in some instances much less.

Much of the true science of this wonderful and humane treatment we have yet to learn. Serious and even discrediting results may develop, but at present we can with conscience recommend cataphoresis in your daily labors.—*Dominion Dental Journal*,

